# Both GPER and membrane oestrogen receptor-α activation protect ventricular remodelling in 17β oestradiol-treated ovariectomized infarcted rats

**DOI:** 10.1111/jcmm.12430

**Published:** 2014-09-25

**Authors:** Tsung-Ming Lee, Shinn-Zong Lin, Nen-Chung Chang

**Affiliations:** a Department of Medicine, Cardiology Section, China Medical University-An Nan HospitalTainan, Taiwan; b Department of Medicine, China Medical UniversityTaichung, Taiwan; c Department of Internal Medicine, School of Medicine, College of Medicine, Taipei Medical UniversityTaipei, Taiwan; d Neuropsychiatry Center, China Medical University HospitalTaichung, Taiwan; e Graduate Institute of Immunology, China Medical UniversityTaichung, Taiwan; f Department of Neurosurgery, China Medical University Beigan HospitalYunlin, Taiwan; g Department of Neurosurgery, China Medical University-An Nan HospitalTainan, Taiwan; h Division of Cardiology, Department of Internal Medicine, Taipei Medical University HospitalTaipei, Taiwan

**Keywords:** echocardiography, gender, hypertrophy, myocardial infarction, remodelling

## Abstract

Clinical and experimental studies have established that gender is a factor in the development of ventricular hypertrophy. We investigated whether the attenuated hypertrophic effect of oestradiol was *via* activation of phosphatidylinositol 3-kinase (PI3K)/Akt/endothelial nitric oxide synthase (eNOS) through non-genomic action. Twenty-four hours after coronary ligation, female Wistar rats were randomized into control, subcutaneous oestradiol treatment or a G-protein coupled oestrogen receptor (GPER) agonist, G-1 and treated for 4 weeks starting from 2 weeks after bilateral ovariectomy. Ventricular hypertrophy assessed by cardiomyocyte size after infarction was similarly attenuated by oestradiol or G-1 in infarcted rats. The phosphorylation of Akt and eNOS was significantly decreased in infarcted rats and restored by oestradiol and G-1, implying the GPER pathway in this process. Oestradiol-induced Akt phosphorylation was not abrogated by G-15 (a GPER blocker). Akt activation was not inhibited by actinomycin D. When a membrane-impermeable oestrogen-albumin construct was applied, similar responses in terms of eNOS activation to those of oestradiol were achieved. Furthermore, PPT, an ERα receptor agonist, activated the phosphorylation of Akt and eNOS. Thus, membrane ERα receptor played a role in mediating the phosphorylation of Akt and eNOS. The specific PI3K inhibitor, LY290042, completely abolished Akt activation and eNOS phosphorylation in infarcted hearts treated with either oestradiol or oestradiol + G-15. These data support the conclusions that oestradiol improves ventricular remodelling by both GPER- and membrane-bound ERα-dependent mechanisms that converge into the PI3K/Akt/eNOS pathway, unveiling a novel mechanism by which oestradiol regulates pathological cardiomyocyte growth after infarction.

## Introduction

Epidemiological studies show that the incidence of cardiovascular disease is higher in men than in premenopausal women but increases in postmenopausal women [1]. The exact pathophysiologic mechanisms by which oestrogen influences cardiac hypertrophy are far from being understood. Oestrogen actions are mediated by a complex interface of direct control of gene expression (genomic action) and by regulation of cell signalling/phosphorylation cascades, referred to as the ‘non-genomic’, or extranuclear, action [2]. In addition to the classical nuclear oestrogen receptor (ER) isoforms ERα and ERβ, G-protein coupled oestrogen receptor (GPER) represents a third ER. GPER has been reported to mediate non-genomic oestradiol (E2) signalling. However, unlike membrane-bound ER, GPER was primarily expressed in the endoplasmic reticulum with no detectable receptor expressed on the cell surface [3]. Thus, there were two distinct pathways mediating non-genomic E2-induced signalling. One is membrane-bound ER, the other GPER. GPER is expressed in both the rodent and human heart, as well as in isolated rat cardiomyocytes [4]. In the acute settings, the GPER specific agonist G-1 reduces infarct size and post-ischaemic contractile dysfunction independent of gender in a model of rat cardiac ischaemia/reperfusion injury [5]. Chronic treatment with G-1 attenuates salt-induced myocyte hypertrophy in the absence of changes in blood pressure [6]. However, the effect of GPER on ventricular remodelling after myocardial infarction (MI) remained unclear.

E2 has been shown to induce phosphoinositide 3-kinase (PI3K)/Akt-dependent phosphorylation of endothelial nitric oxide synthase (eNOS) in the post-ischaemic kidney and in cerebral vessels [7,8]. However, the effect of PI3K/Akt/eNOS axis on E2-induced cardiac remodelling remains unclear. PI3K activity is essential for maladaptive pathological myocardial hypertrophy [9]. Cardiac specific overexpression of constitutively active PI3K in mice results in enlarged hearts owing to increased cardiomyocyte size. Akt was identified as a downstream component of survival signalling through PI3K [10]. Activated Akt phosphorylates several downstream targets including eNOS [11]. Knockout of Akt attenuates normal physiological growth while promoting pathological myocardial hypertrophy [12,13].

Cardiac remodelling is an unfavourable evolution associated with myocardial hypertrophy, fibrosis and ventricular dysfunction after MI [14]. Cardiac remodelling is a complex process involving numerous signalling pathways. Pharmacological therapies, such as angiotensin-converting enzyme inhibitors and β-blockers through different molecular targets, have been used to improve cardiac remodelling [15]. We have previously shown that LV remodelling is improved after E2 treatment most likely through an endothelin-1 dependent pathway after infarction [16]. However, in other studies oestrogen either offered no cardioprotective effect or even worsened cardiac remodelling and dysfunction post-MI [17,18]. Thus, the cardiac effect of oestrogen appears to be more complicated than originally thought and requires further research. The precise mechanism by which E2 affected cardiac remodelling remains unknown. Thus, we assessed (*i*) whether adjunctive administration of physiological concentrations of E2 after infarction can result in attenuated ventricular remodelling, (*ii*) whether the PI3K/Akt/eNOS axis plays a role in mediating remodelling, and (*iii*) whether GPER and membrane ERα modulate the activities of eNOS in a rat MI model.

## Materials and methods

### Animals

All rats received humane care and the experiment was approved and conducted in accordance with local institutional guidelines of the China Medical University for the care and use of laboratory animals (IACUC, Permit Number: 102-63-N) and conformed to the *Guide for the Care and Use of Laboratory Animals* published by the US National Institutes of Health (NIH Publication, 8th Edition, 2011).

#### Experiment 1 (*in vivo* study)

Female Wistar rats that weighed 120–150 g (around 8 weeks old) received ovariectomies (OVX) through dorsal incisions under anaesthesia with ketamine–xylazine (90 mg/kg–9 mg/kg, intraperitoneally). The anaesthesia of rats was considered sufficient when the paw pinch was negative. Two weeks after OVX, the rats were subjected to permanent ligation of the anterior descending artery as previously described [16] resulting in infarction of the LV free wall. Twenty-four hours after inducing MI, OVX rats were randomly assigned into three groups so as to have approximately the same number of survivors in each group (Table 1): (*i*) control group (DMSO/EtOH), (*ii*) E2 (0.5 mg, subcutaneous 60-day release pellets, Innovative Research of America, Sarasota, Florida) or (*iii*) G-1 (50 μg/kg per day in a DMSO/EtOH mixture, Cayman Chemical Company, Ann Arbor, MI, USA). Sham group rats underwent the same surgical procedures except for ligation. We have previously demonstrated that E2, administered prior to coronary ligation, can provide cardioprotection against infarct size and arrhythmias [19]. To avoid the confounding effect, the E2 pellets were implanted subcutaneously in the neck area at 24 hrs after MI. The sustained release pellets were designed to maintain constant E2 concentrations instead of the fluctuating E2 levels that occur in intact rats during the oestrous cycle. Confirmation of oestrogen status was determined by uterus weight and plasma E2 levels. The control OVX rats without treatment with E2 pellets received placebo pellets. The dose of G-1 has been shown to abolish GPER signalling without hemodynamic changes [6]. The drugs were given orally by gastric gavage once a day. In each treated group, drugs were withdrawn ∼24 hrs before the end of the experiments to eliminate their pharmacological actions. Thus, together, 8 experimental groups were studied: sham groups (intact without OVX, OVX/control treated with DMSO/EtOH, OVX/E2, OVX/G-1), and infarction groups (intact without OVX, OVX/control treated with DMSO/EtOH, OVX/E2, OVX/G-1). The rats in the intact group were not OVX and thus had a functional endogenous E2 level (intact group). The rats in OVX/control group were OVX and treated with placebo pellets and DMSO/EtOH. The rats in the OVX/E2 group were OVX and treated with E2 pellets. The rats in the OVX/G1 group were OVX and treated with G1 (Supporting Information). The study duration was designed to be 4 weeks because the majority of the myocardial remodelling process in the rat (70–80%) is complete within 3 weeks [20].

**Table 1:** Morphometry, haemodynamics, E2 concentrations and echocardiographic findings at the end of the study

		Infarcted rats treated with			
Parameters	Sham	Intact	OVX/control	OVX/E2	OVX/G-1
No. of rats	10	9	8	8	7
Bodyweight (g)	242 ± 12	245 ± 14	286 ± 16*	250 ± 12	245 ± 15
HR (bpm)	411 ± 15	410 ± 14	435 ± 12*	423 ± 11	425 ± 15
LVESP (mmHg)	98 ± 7	95 ± 7	105 ± 10	95 ± 8	92 ± 6
LVEDP (mmHg)	5 ± 2	19 ± 2†	17 ± 4†	16 ± 4†	15 ± 4†
Heart W/BW (mg/g)	4.71 ± 0.10	4.88 ± 0.20	4.57 ± 0.21	4.62 ± 0.18	4.75 ± 0.24
LVW/BW (mg/g)	3.22 ± 0.15	3.35 ± 0.32	3.20 ± 0.26	3.14 ± 0.22	3.33 ± 0.32
RVW/BW (mg/g)	0.57 ± 0.11	0.74 ± 0.12†	0.75 ± 0.12†	0.69 ± 0.13†	0.65 ± 0.15
+dp/d*t* (mmHg/sec.)	7655 ± 219	3094 ± 232†	4652 ± 236*	3281 ± 236†	3188 ± 245†
-dp/d*t* (mmHg/sec.)	5622 ± 174	2156 ± 243†	3022 ± 215*	2082 ± 205†	2382 ± 256†
Infarct scar size (% of LV)	…	41 ± 2	40 ± 2	41 ± 2	40 ± 2
Uterus weight (mg/g BW)	62 ± 9	87 ± 12	21 ± 14†,‡	92 ± 15†	31 ± 15†
E2 (pg/ml)	74 ± 16	78 ± 14	17 ± 12†,‡	88 ± 20	17 ± 14†
LVEDD (mm)	5.1 ± 0.2	8.4 ± 0.5†	8.3 ± 0.4†	8.5 ± 0.6†	8.3 ± 0.5†
LVESD (mm)	3.0 ± 0.2	7.0 ± 0.5†	6.9 ± 0.4†	7.0 ± 0.5†	7.0 ± 0.5†
FS (%)	42 ± 2	17 ± 4†	17 ± 4†	18 ± 5†	16 ± 5†

* *P* < 0.05 compared with sham and infarcted groups treated with intact, OVX/E2, and OVX/G-1.

† *P* < 0.05 compared with sham group.

‡ *P* < 0.05 compared with sham and infarcted groups treated with intact and OVX/E2.

Values are mean ± sd.

BW, bodyweight; OVX/control, ovariectomy treated with vehicle; E2, estradiol; FS, fractional shortening; HR, heart rate; LVEDD, left ventricular end-diastolic dimension; LVEDP, left ventricular end-diastolic pressure; LVESD, left ventricular end-systolic dimension; LVESP, left ventricular end-systolic pressure; LVW, left ventricular weight; RVW, right ventricular weight.

#### Experiment 2 (*ex vivo* study)

Although results of the above study showed that the activities of Akt and eNOS were significantly increased after administering E2 (see Results), the involved mechanism remained unclear. To test the importance of GPER and PI3K in E2-related Akt and eNOS activities, we used ICI 182,780 (a nuclear ERs blocker), actinomycin D (an inhibitor of gene transcription) and LY294002 (a PI3K inhibitor) in an *ex vivo* model. Four weeks after the induction of MI by coronary ligation in female OVX Wistar rats, infarcted hearts were isolated and subjected to control, E2 (1 nM; Sigma-Aldrich), G-1 (1 nM; Cayman Chemical Company), E2+ ICI 182,780 (100 nM; Sigma-Aldrich), E2+ actinomycin D (5 μg/ml; Sigma-Aldrich), or E2+ LY294002 (50 μM, 2-(4-morpholinyl)-8-phenyl-4*H*-1-benzopyran-4-one; Sigma-Aldrich). Each heart was perfused with a non-circulating modified Tyrode's solution containing (in mM): NaCl 117.0, NaHCO_3_ 23.0, KCl 4.6, NaH_2_PO_4_ 0.8, MgCl_2_ 1.0, CaCl_2_ 2.0 and glucose 5.5, equilibrated at 37°C and oxygenated with a 95% O_2_ to 5% CO_2_ gas mixture. The drugs were perfused for 30 min. The doses of E2 [21], G-1 [22], ICI 182,780 [21], actinomycin D [21] and LY294002 [21] have been shown to effectively modulate biological targets. At the end of the study, all hearts (*n* = 5 each group) were used for performing Western blot for Akt and eNOS at the remote zone.

#### Experiment 3 (*ex vivo* study)

To specifically assess the role of membrane ERα and PI3K in activating Akt and eNOS, we employed E2-BSA (E2 conjugated with bovine serum albumin, a membrane-impermeable conjugate of E2), G-15 (a specific GPER blocker) and PPT (4,4′,4″-(4-propyl-(1H)-pyrazole-1,3,5-triyl)trisphenol, a specific ERα agonist). Four weeks after the induction of MI by coronary ligation in female OVX Wistar rats, infarcted hearts were isolated and subjected to control, E2-BSA (33 mol E2/mol BSA), E2+ G-15 (10 nM, Tocris Bioscience), E2+ G-15+ LY294002 (50 μM; Sigma-Aldrich), PPT (10 nM; Tocris Bioscience, Ellisville, MI, USA), or PPT + LY294002. Each heart was perfused as the protocol of Part 2 and the drugs were perfused for 30 min. The doses of G-15 [22], and PPT [22] have been shown to effectively modulate biological targets. At the end of the study, all hearts (*n* = 5 each group) were used for performing Western blot for Akt and eNOS at the remote zone.

### Experimental MI

After anaesthesia with ketamine–xylazine (90 mg/kg–9 mg/kg, intraperitoneally), rats were intubated and the left anterior descending artery was ligated using a 6-0 silk as previously described [16]. Sham rats underwent the same procedure except the suture was passed under the coronary artery and then removed. Mortality in the animals with MI was ∼50% within the first 24 hrs according to our experience. None of the sham-operated animals died.

### Echocardiogram

At 28 days after operation, rats were lightly anesthetized with an intraperitoneal injection of ketamine–xylazine (45 mg/kg–5 mg/kg). Echocardiographic measurements were done with a HP Sonos 5500 system with a 156L (6–15 MHz, SONOS 5500; Agilent Technologies, Palo Alto, CA, USA) probe. M-mode tracing of the LV was obtained from the parasternal long-axis view to measure LV end-diastolic diameter dimension and LV end-systolic diameter dimension, and fractional shortening (FS) (%) was calculated. After this, the rats quickly underwent hemodynamic measurement after systemic heparinization.

### Haemodynamics and infarct scar size measurements

Using a 2F micromanometer-tipped catheter (Model SPR-407; Millar Instruments, Houston, TX, USA) inserted through the right carotid artery as our previous description [16], we measured the maximal rate of LV pressure rise (+dP/d*t*) and decrease (−dP/d*t*). Next, the heart was rapidly excised and divided into right and left atria, right ventricles, LV, and the scarred area. Each tissue was then weighed individually. Infarct scar size (%) was expressed as the ratio of the sum of external and internal perimeters of LV as described by Pfeffer *et al*. [23]. Samples of the LV from the remote zone were cut transmurally, frozen rapidly in liquid nitrogen, and stored at −80°C until use.

### Western blot analysis of ser473-phosphorylated Akt1, Akt1, Ser1177-phosphorylated eNOS and eNOS

Samples obtained from the remote zone were homogenized with a kinematic polytron blender in 100 mm Tris HCl (pH 7.4), supplemented with 20 mmol/litre EDTA, 1 mg/ml pepstatin A, 1 mg/ml antipain and 1 mmol/l benzamidin. Homogenates were centrifuged at 10,000 × g for 30 min. to pellet the particulate fractions. The supernatant protein concentration was determined with the BCA protein assay reagent kit (Pierce Endogen, Rockford, IL, USA). Twenty micrograms of protein were separated by 10% SDS-PAGE and electrotransferred onto a nitrocellulose membrane. After incubation with antibodies, antigen-antibody complexes were detected with 5-bromo-4-chloro-3-indolyl-phosphate and nitroblue tetrazolium chloride (Sigma-Aldrich). Films were volume-integrated within the linear range of the exposure using a scanning densitometer. Experiments were replicated three times and results expressed as the mean value.

The primary antibodies used were as follows: p-Akt1 (ser473; Cell Signaling Technology, Beverly, MA, USA), Akt1 (Santa Cruz Biotechnology, Santa Cruz, CA, USA), p-eNOS (ser1177; Cell Signaling Technology), eNOS (Cell Signaling Technology) and β-actin (Santa Cruz Biotechnology).

### Morphometric determination of myocyte size and fibrosis

Because ventricular remodelling after infarction is a combination of myocyte hypertrophy and reactive fibrosis, we measured cardiomyocyte sizes and fibrosis in addition to myocardial weight to avoid the confounding influence of non-myocytes on cardiac hypertrophy. LV sections from the remote zone were stained with haematoxylin and eosin. For consistency of results, myocytes positioned perpendicularly to the plane of the section with a visible nucleus and cell membrane clearly outlined and unbroken were then selected for the cross-sectional area measurements [24]. This area was determined by manually tracing the cell contour on a digitized image acquired on the image-analysis system at a magnification of 400 using computerized planimetry (Image Pro Plus) as previously described [25]. A total of 100 myocytes were selected in the LV of each heart and analysed by an observer blinded to the experimental treatment.

Additionally, heart sections were stained with Sirius red stain to distinguish areas of connective tissue as previously described [24]. The percentage of red staining, indicative of fibrosis, was measured (10 fields randomly selected on each section). The value was expressed as the ratio of Sirius red-stained fibrosis area to total area. All sections were evaluated under blinded conditions without prior knowledge as to which section belonged to which rat.

### Measurement of plasma E2

Blood samples (1 ml) were withdrawn from the LV into EDTA-coated tubes for plasma samples. Blood samples were separated by centrifugation at 3000 × g for 10 min. at 4°C. E2 levels were measured with the use of a RIA kit (Diagnostic Products Corp., Los Angeles, CA, USA).

### Statistical analysis

Results were presented as mean ± SD. Statistical analysis was performed with the spss statistical package (SPSS, version 12.0, Chicago, IL, USA). Differences among the groups were tested by anova. In cases where a significant effect was observed, the measurements between the groups were compared, with *P* < 0.05 (Bonferroni's correction) as significant. Correlation between cardiomyocyte size and the eNOS levels was assessed using Pearson's correlation coefficient. Probability values were two-tailed, and *P* < 0.05 was considered to be statistically significant.

## Results

### Part 1 *in vivo* study

**Fig. 1:**
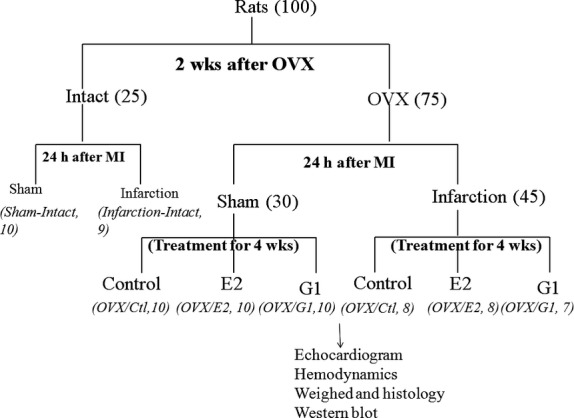
Flow chart. In terms of ovariectomy (OVX), rats were divided into intact and OVX. In terms of induced myocardial infarction, rats were divided into sham (receiving sham operation) and infarction. In terms of treatment, rats were divided into control (receiving placebo pellets), E2 (0.5 mg, subcutaneous 60-day release pellets) or G1. Thus, together, the experimental groups studied were: sham groups (intact, OVX/control, OVX/E2, OVX/G1) and infarction groups (intact, OVX/control, OVX/E2, OVX/G1). The number of animals in each group is indicated in parentheses.

Differences in mortality among the infarcted groups were not found throughout the study (Fig. 1). Four weeks after infarction, the infarcted area of the LV was very thin and was totally replaced by fully differentiated scar tissue. Mean infarct scar size was similar among the infarcted groups (Table 1). The weight of the LV inclusive of the septum remained essentially constant for 4 weeks among the infarcted groups.

#### Bodyweight, E2 levels and uterus weight

Bodyweight was significantly higher in infarcted rats treated with control OVX (Table 1) despite there being no difference in weight among any of the groups at the start of the study. The finding that bodyweights were negatively related to E2 status was consistent with prior observations [26]. Interestingly, G-1 treatment attenuated the effect of ovarian hormone loss on bodyweight. MI did not affect bodyweight.

Ovariectomies animals replaced with an E2 pellet had an average plasma E2 level similar to that seen at proestrus in the cycling rat (average 88 ± 20 pg/ml). In contrast, OVX/control animals had low levels of oestrogen (average 17 ± 12 pg/ml), similar to that found in postmenopausal women.

Uterine weight was used as an indicator for effectiveness of OVX. The weights of the uterine horns and body were significantly less in OVX/control than sham-operated rats (*P* < 0.0001, Table 1). There was a significant increase in uterine weight in OVX rats treated with E2 pellets than sham. G-1 treatment significantly decreased uterine weight, as compared to rats treated with E2. MI had no effect on uterus weight.

#### Haemodynamics

In sham rats, LV end-diastolic pressure, LV end-systolic pressure, +dP/d*t* and −dP/d*t* were not affected by E2 status (data not shown). Heart rate was significantly higher in infarcted rats treated with OVX/control than in the rats treated with intact, OVX/E2, and OVX/G-1 (Table 1). LV end-diastolic pressure was similarly increased in the infarcted groups compared with sham.

#### Echocardiographic data

**Fig. 2:**
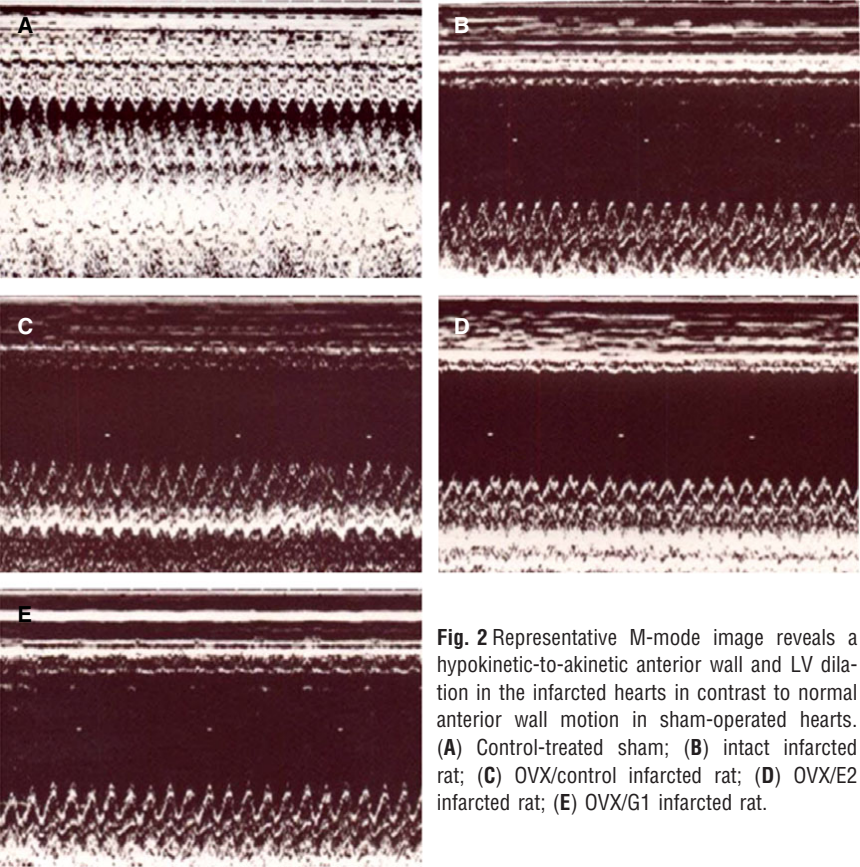
Representative M-mode image reveals a hypokinetic-to-akinetic anterior wall and LV dilation in the infarcted hearts in contrast to normal anterior wall motion in sham-operated hearts. (**A**) Control-treated sham; (**B**) intact infarcted rat; (**C**) OVX/control infarcted rat; (**D**) OVX/E2 infarcted rat; (**E**) OVX/G1 infarcted rat.

After 4 weeks of E2 intervention, LV structure and function were evaluated *in vivo* by echocardiographic analysis (Table 1, Fig. 2). When compared with sham-operated hearts, infarcted hearts showed structural changes such as increased LV diastolic and systolic diameters. The combination of impaired regional function and LV cavity enlargement resulted in a substantial decrease in FS (42 ± 2% in sham *versus* 17 ± 4% in intact infarcted hearts; *P* < 0.0001). Modulation of hormonal status by OVX, E2 or drug intervention by G-1 had no effect on echocardiographic indices of cardiac function in infarcted hearts.

#### Myocyte size and fibrosis

**Fig. 3:**
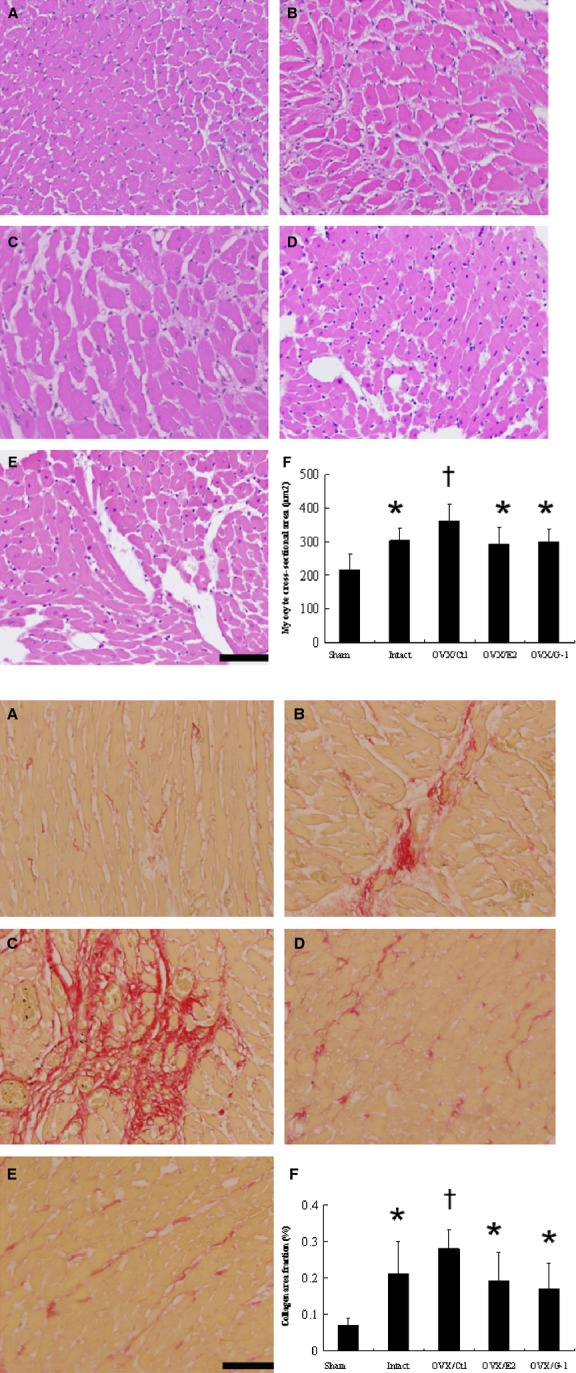
Upper panel: Quantitative analysis of the cardiomyocyte sizes (magnification 400×). Lower panel: Representative sections from the remote zone with Sirius Red staining (red, magnification 400×) at 4 weeks after infarction. (**A**) Control-treated sham; (**B**) intact infarcted rat; (**C**) OVX/control infarcted rat; (**D**) OVX/E2 infarcted rat; (**E**) OVX/G1 infarcted rat. (**F**) Each column and bar represents mean ± SD (*n* = 5–6 each group); bar = 50 μm. *P < 0.05 compared with sham and OVX/control infarcted rats; †*P* < 0.05 compared with sham.

To characterize the cardiac hypertrophy on a cellular level, morphometric analyses of LV sections from the remote zone were performed on different treatment groups (Fig. 3 upper panel). Myocytes were significantly hypertrophied in the intact infarcted group compared with those in the sham-operated group. Furthermore, OVX/control infarcted rats had a further increase in cardiomyocyte size compared with intact infarcted rats. E2 supplementation and G-1 reduced cell areas by 18.5% and 16.9% compared with the OVX/control infarcted rats (all *P* < 0.05).

Fibrosis of the LV from the remote zone was examined in tissue sections after Sirius red staining, as shown in Figure 3 lower panel. Intact infarcted rats had significantly larger areas of intense focal fibrosis compared with sham-operated rats (*P* < 0.05). When compared with intact infarcted rats, OVX/control infarcted rats had furthermore increased fibrosis, as observed by increased collagen staining. The OVX infarcted rats showed attenuated cardiac fibrosis after administering either E2 or G-1.

#### Effects of E2 and G-1 on Akt and eNOS

**Fig. 4:**
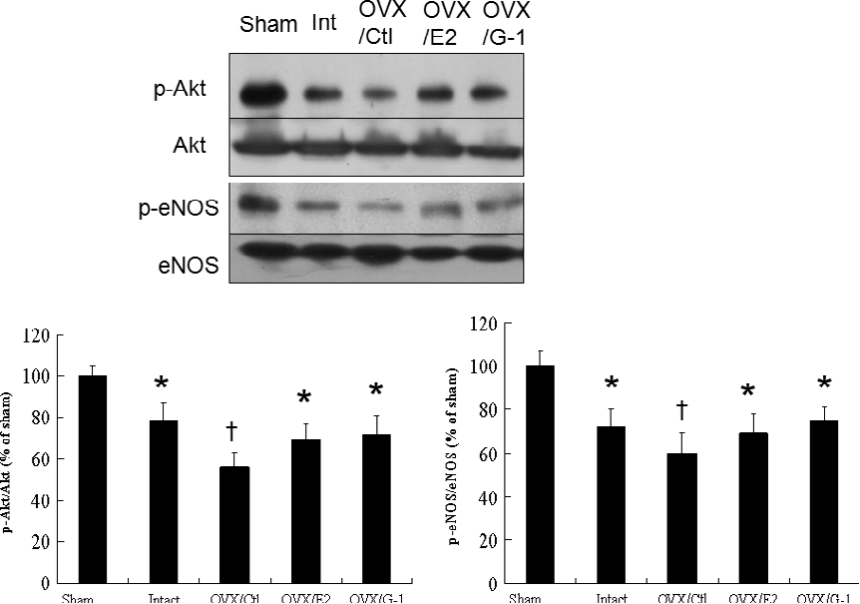
Experiment 1, an *in vivo* study. Representative Western blots show the levels of p-Akt (ser473), Akt, p-eNOS (ser1177) and eNOS. Bar graphs represent the quantitative analysis and difference in the levels of p-Akt (ser473) and p-eNOS (ser1177) after they are normalized with corresponding total proteins, respectively, in % of sham. The values are mean ± SD (*n* = 5–6 each group). Ctl, control. *P < 0.05 compared with sham and OVX/control infarcted rats; †*P* < 0.05 compared with sham.

As eNOS is a physiological substrate for Akt in cardiomyocytes [27], we assessed whether E2-induced Akt activation results in increased eNOS phosphorylation in hypertrophied cardiomyocytes after infarction. As shown in Figure 4, OVX, E2, and G-1 replacement do not affect the total Akt level. However, Akt activation, presented as a p-Akt/Akt ratio, was significantly reduced in the OVX/control infarcted group compared with the intact infarcted group and restored by E2 or G-1 replacement. Similar to the reduced Akt activity, we also found a significant reduction of eNOS activity in the OVX/control infarcted group compared with the intact infarcted group. The linear regression models showed that significant negative linear correlation was observed between the cardiomyocyte sizes and LV eNOS levels (*r* = 0.452, *P* < 0.01). Taken together, these observations show that either OVX or infarction showed a marked decrease with regards to Akt and eNOS activity; however, both can be reversed after adding E2 or G1.

### Part 2 *ex vivo* study

#### Effects of GPER on E2-treated Akt and eNOS activities

**Fig. 5:**
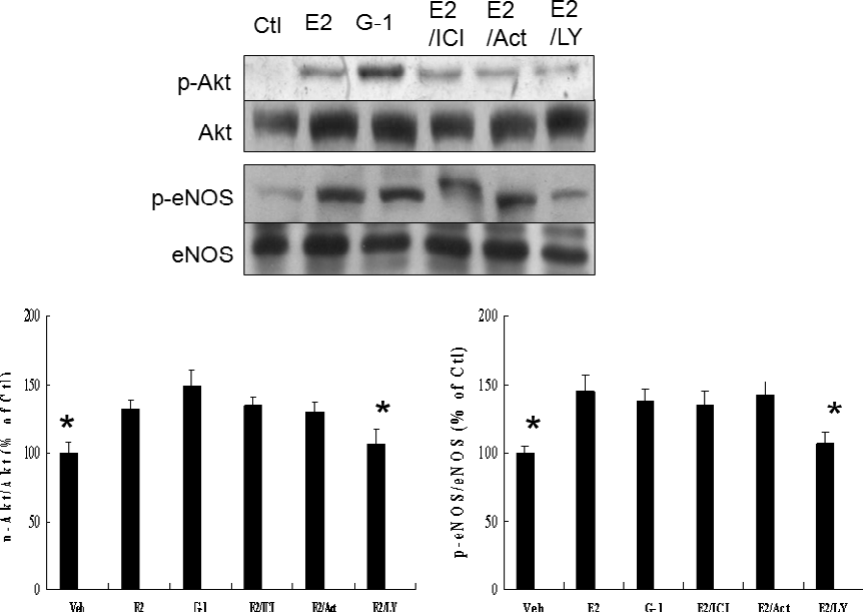
Experiment 2, an *ex vivo* study to assess effects of GPER on E2-treated Akt and eNOS activities. Western blot analysis of Akt and eNOS to confirm the GPER on enzyme activity in homogenates of the LV from the remote zone in a rat isolated infarcted heart model. E2 administration shows similarly increased phosphorylation of Akt and eNOS compared with G-1. Either ICI 182,780 (ICI) or actinomycin D (Act) does not affect the induction of Akt and eNOS activities by E2. Bar graphs represent the quantitative analysis and difference in the levels of p-Akt (ser473) and p-eNOS (ser1177) after they are normalized with corresponding total proteins, respectively, in % of E2 group. The values are mean ± SD (*n* = 5 each group). Ctl, control; LY, LY294002. *P < 0.05 compared with E2, G-1, E2+ ICI, and E2+ Act.

In this *ex vivo* study, Western blot revealed that E2 administration showed similarly increased phosphorylation of Akt and eNOS compared with G-1, a finding similar to *in vivo* results. To determine whether this process involves rapid ER activation, the effect of concomitant treatment with the pure ER antagonist ICI 182,780 was determined (Fig. 5). ICI-182,780 did not decrease E2-induced Akt activation. Moreover, actinomycin D, an inhibitor of gene transcription, was used to rule out the influence of genomic events mediated by nuclear ERs. Actinomycin D did not affect the induction of Akt activity by E2. Intriguingly, addition of LY294002, a PI3K inhibitor, not only suppresses the phosphorylation of Akt but also down-regulates p-eNOS (ser1177). These results suggest that enhanced Akt activity and concomitant eNOS phosphorylation by E2 treatment are likely mediated by non-genomic GPER-induced PI3K/Akt -dependent pathway.

#### Effects of membrane ERα on E2-treated Akt and eNOS activities

**Fig. 6:**
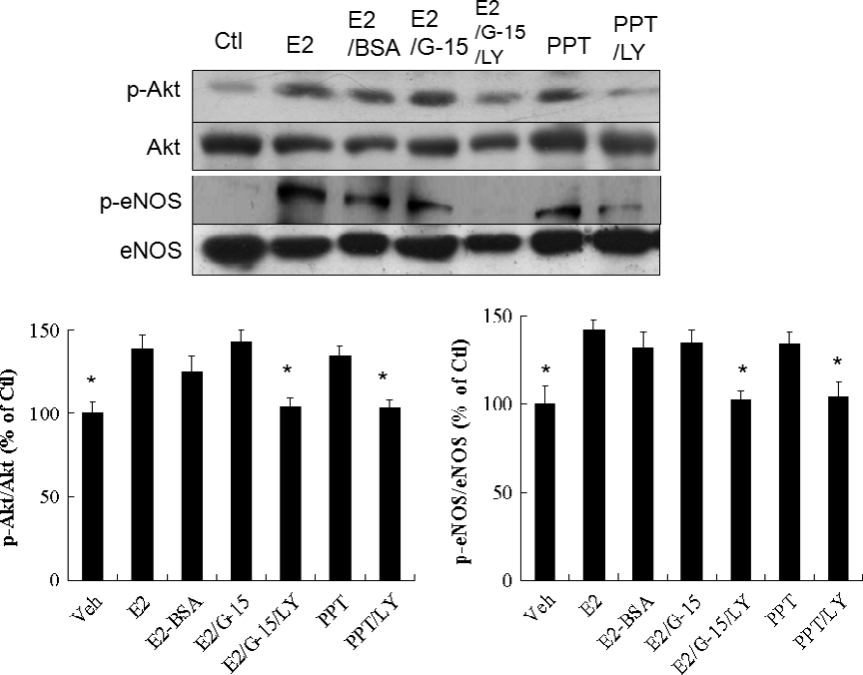
Experiment 3, an *ex vivo* study to assess effects of membrane ERα on E2-treated Akt and eNOS activities. E2-BSA administration shows similarly increased phosphorylation of Akt and eNOS compared with E2. G-15 does not affect the induction of Akt and eNOS activities by E2. Bar graphs represent the quantitative analysis and difference in the levels of p-Akt (ser473) and p-eNOS (ser1177) after they are normalized with corresponding total proteins, respectively, in % of E2 group. The values are mean ± SD (*n* = 5 each group). Ctl, control; LY, LY294002. *P < 0.05 compared with E2, E2-BSA, E2+ G-15 and PPT.

To elucidate the role of E2-related membrane ERα in modulating Akt and eNOS activities, G-15 was assessed in an *ex vivo* model (Fig. 6). Surprisingly, G-15 did not abolish the E2-induced Akt activation, implying an alternative pathway to activate Akt except GPER. To assess the potential role of membrane-bound ERα induced Akt activation, we employed a membrane ERα agonist, PPT. PPT treatment alone activates Akt activity. Furthermore, E2–BSA, a membrane-impermeable conjugate of E2, also stimulated an increase in Akt activity. Co-infusion with LY294002 not only down-regulated the levels of phosphorylated Akt, but also down-regulated eNOS phosphorylation in the infarcted hearts treated with E2+ G-15, which suggested that the PI3K/Akt signal pathway participated in modulating the activity of eNOS.

## Discussion

We investigated the molecular mechanisms underlying the inhibitory effects of E2 on ventricular remodelling in infarcted rats. E2 attenuated post-infarcted LV hypertrophy and fibrosis, an effect similar to chronic GPER activation by its agonist G-1, independent of changes in blood pressure. One striking and novel finding from our study was that both membrane-bound ERα and GPER directly and independently regulated eNOS, with convergent results, including enhanced PI3K and increased Akt activity. These results were consistent with the beneficial effects of E2, as documented structurally by the reduction in cardiomyocyte sizes and cardiac fibrosis, molecularly, by myocardial Akt and eNOS activities, and pharmacologically by G-1, G-15 and LY294002 administration. The dual and not exclusive pathways to activate eNOS may explain the mixed results of previous studies.

**Fig. 7:**
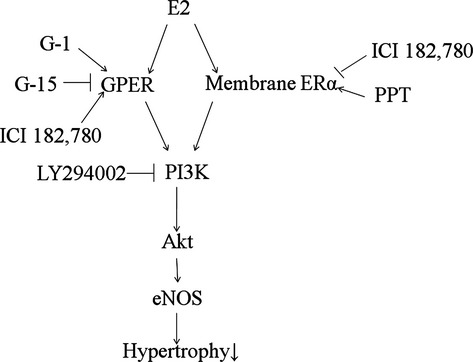
Reaction sequences leading to attenuated cardiomyocyte hypertrophy after infarction. The diagram summarizes the anatomical, molecular and pharmacological evidence presented in this report. Inhibition of these signalling pathways by their respective inhibitors is indicated by the vertical lines.

The present study illustrates an additional role for E2, showing that both GPER and membrane-bound ERα signallings are operative to improve ventricular remodelling through a common PI3K/Akt/eNOS-dependent pathway. The beneficial effect of E2 on the ventricular remodelling was supported by the following evidence (Fig. 7):

1. E2 stimulation can augment Akt and eNOS activities through a GPER-mediated pathway. GPER has been shown to be present in cardiomyocytes [28]. E2 increased the phosphorylation of Akt and eNOS that was not reversed by ICI 182,780, supporting an ER-independent mechanism. In this study, the non-steroidal receptor antagonist ICI 182,780 was added at a concentration much greater (100 nM) than that of E2 (1 nM), a dose relationship that effectively blocks classic receptor-mediated oestrogenic effects [29]. ICI 182,780 is an efficacious antagonist of the ER, but it is also a full agonist for GPR30 [30]. Thus, studies examining the ability of ICI 182,780 to mimic or antagonize Akt activities may enable pharmacological discrimination that may be mediated *via* ERs *versus* GPER. Akt activities mediated by ERs would be expected to be blocked by ICI 182,780; effects mediated by GPER would be expected to be increased by ICI 182,780. The effect of ICI 182,780 to increase Akt activities provided an index for responses exhibiting high GPER sensitivity.
2. E2 stimulation can augment Akt and eNOS activities through a membrane-bound ERα-mediated pathway. The acute response of Akt and eNOS to E2 was not inhibited by concomitant treatment with the GPER antagonist G-15. Furthermore, the ability of membrane-impermeable E2-BSA to rapidly activate Akt signalling also indicates that rapid oestrogenic effects are initiated at the plasma membrane. There was an apparent increase in Akt activity upon brief stimulation with PPT, a membrane ERα activator. Furthermore, the rapidity of the activation of Akt and eNOS by E2 along with the fact that the activation was not altered by the inhibition of gene transcription by actinomycin D indicate that the process may not require the classical nuclear effects of the oestrogen. These results suggested that an E2-induced PI3K/Akt cascade stimulates the activation of eNOS through a membrane receptor. Overall, our data were consistent with the notion that E2-induced endothelial NO release within minutes is ER-dependent and gene transcription-independent [31]. Our results were consistent with the findings of Hisamoto *et al*. [32], showing that E2 induces Akt activity through membrane-bound ERα.
3. Although receptors for E2 eliciting antihypertrophic effects are distinct, elevated PI3K/Akt/eNOS signalling is probably a common pathway to rescue the heart from hypertrophy. Both pathways of GPER and membrane ERα converge into the common signalling. The E2-triggered activation induces rapid phosphorylation of the critical serine 473 Akt residue and consequent activating phosphorylation of serine 1177 on eNOS. The relevance of this activation is confirmed by abrogation of E2-stimulated nitric oxide release with pharmacological inhibition of PI3K. Our results were consistent with previous findings, showing that inhibition of the PI3K/Akt pathway or mutation of the Akt site on eNOS protein (at serine 1177) attenuates the serine phosphorylation and prevents the activation of eNOS [33].

Our results showed that Akt and eNOS play a pivotal role to protect the heart from pathological hypertrophy and they are consistent with the findings, showing that Akt1^−/−^ mice develop more profound hypertrophy after transverse aortic constriction than do wild-type mice [12] and eNOS-deficient mice showed marked increase in LV mass [34].

### Previous studies

Previous studies have shown that in OVX female mice, E2 supplementation 7 days after OVX resulted in increased cardiomyocyte hypertrophy in infarcted mice [35], contrast with our results. The difference for this discrepancy is unclear; however, different species (mice *versus* rats), supplementation timing (7 days *versus* 15 days) and E2 concentrations (pharmacological *versus* physiological) may play a role. The cardiovascular effects of hormone replacement therapy include both beneficial and detrimental effects. First, the timing of E2 supplementation is an important issue. Markedly reduced oestrogen in postmenopausal women is associated with increased morbidity and death from cardiovascular diseases [1]. Even in premenopausal women, bilateral oophorectomy is associated with increased cardiovascular risk, and oophorectomy at a young age further increases this risk [36]. Oestrogen therapy begun early after surgical or natural menopause at a young age seems to reduce this risk [36]. However, prolonged deprivation of oestrogen may result in a loss of assembled signal transduction complexes [37], rendering the heart insensitive to future oestrogen administration. Second, previous studies have shown that at a physiological dose, E2 tended to improve cardiac function and remodelling after infarction, but at a pharmacological dose, E2 exacerbated cardiac hypertrophy, dysfunction and remodelling [38]. Indeed, our data are in agreement with the finding of Pedram *et al*. [39], showing that E2 at a physiological dose attenuated cardiomyocyte hypertrophy.

### Clinical implications

After acute MI, patients remain at high risk for recurrent cardiovascular events and mortality. Improvement in post-infarct ventricular remodelling is not a negligible problem in the clinical areas. We provide *in vivo* evidence for a role of E2 after infarction that enables regulation of cardiomyocyte hypertrophy in live animals. The interventions that activate the PI3K/Akt/eNOS signalling pathway may have therapeutic benefits for post-MI-associated complications.

Gender studies of the cardiovascular system have focused on oestrogen's vasoprotective effects; however, it is now obvious that oestrogen has direct effects on the myocardium independent of its vasoprotective effect. Our findings point towards another potential oestrogen-mediated cardioprotective mechanism involving a distinct pathway from that of classical nuclear ERs. Moreover, these observations provide a basis for the generation of novel therapeutic approaches at the cardiac level. In the specific setting of acute MI, there is evidence that oestrogen may improve mortality [40]. The controversial outcome of clinical trials may partly result from the complexity of oestrogen signalling, which involves at least three different ERs. The question therefore remains how to pharmacologically separate the beneficial signalling pathways of oestrogens from their harmful actions. With accumulating evidence that GPER is responsible for a variety of beneficial cardiovascular effects of oestrogens, this receptor may represent a novel target to develop effective strategies for the treatment of cardiovascular diseases by tissue-specific, selective activation of oestrogen-dependent molecular pathways devoid of side effects seen with conventional hormone therapy. Our findings indicate that G-1 is a novel extranuclear selective ER modulator *in vivo* and provides cardiovascular protection without stimulating uterine growth.

### Study limitations

There are limitations in the translation of the results of this study to other species and to human physiology that should be highlighted. First, only one time-point after infarction was studied. The cardiac fibrosis was not assessed until 4 weeks after infarction. It is known that after MI, the infarcted region of the heart undergoes a dynamic healing and repair process. The process of scar formation takes 3 weeks in the rat and involves the deposition of extracellular matrix, which serves to limit infarct expansion [23]. E2 has been shown to inhibit the growth of cardiac fibroblasts [41]. The suppression of fibroblast growth and collagen synthesis may be in part responsible for the increased infarct scar size found in the E2-treated rats at 2 weeks after infarction [42], which was not consistent with our results observed at 4 weeks after infarction. This discrepancy was explained, at least in part, by the fact that there are numerous potential mechanisms whereby E2 exerts beneficial effects at a chronic stage of MI. For example, E2 acts as a potent vasodilator, through up-regulation of vasodilator pathways, such as eNOS. Future research should identify these early events and trace their progression after administering E2 after infarction. Second, E2-BSA has been widely used as a membrane-impermeable form of oestrogen to test for the presence of cell-surface ERs. However, E2-BSA contains an amount of free immunoassayable E2, E2-BSA binds to ER only poorly because the E2 is linked to BSA through groups that are important for ER binding, and certain E2-BSA preparations are of very high molecular weight, suggesting extreme protein cross-linking [43]. Second, in menopause the depletion of oestrogen is a slow and progressive process, whereas surgically induced OVX causes a sudden depletion. Early OVX in females does not address age-related changes that influence the cardiac hypertrophy of older women. Recent studies have shown that ageing is an important factor that modulates PI3K/Akt activation [44]. Thus, a more hormonally relevant model mimicking human menopause might be useful in future studies.

## Conclusions

The beneficial ventricular remodelling afforded by oestrogen is likely mediated by effects on both GPER and membrane-bound ER signalling pathways. Both pathways have been shown to converge in activation of PI3K/Akt and increased phosphorylation and activity of eNOS. These observations open a new therapeutic perspective for intervention in the hypertrophic process and, at the same time, the modulation of the GPER and membrane-bound ER signalling may provide novel therapeutic targets for which a new class of antihypertrophic drugs can be designed.
